# Inappropriate Intra-cervical Injection of Radiotracer for Sentinel Lymph Node Mapping in a Uterine Cervix Cancer Patient: Importance of Lymphoscintigraphy and Blue Dye Injection

**Published:** 2014

**Authors:** Sima Kadkhodayan, Elham Hosseini Farahabadi, Zohreh Yousefi, Malihe Hasanzadeh, Ramin Sadeghi

**Affiliations:** 1 Women's Health Research Center, Mashhad University of Medical Sciences, Mashhad, Iran; 2 Nuclear Medicine Research Center, Mashhad University of Medical Sciences, Mashhad, Iran

**Keywords:** ^99m^Tc-Phytate, Cervical cancer, Lymphoscintigraphy, Methylene blue, Sentinel node

## Abstract

Herein, we report a case of sentinel lymph node mapping in a uterine cervix cancer patient, referring to the nuclear medicine department of our institute. Lymphoscintigraphy images showed inappropriate intra-cervical injection of radiotracer. Blue dye technique was applied for sentinel lymph node mapping, using intra-cervical injection of methylene blue. Two blue/cold sentinel lymph nodes, with no pathological involvement, were intra-operatively identified, and the patient was spared pelvic lymph node dissection. The present case underscores the importance of lymphoscintigraphy imaging in sentinel lymph node mapping and demonstrates the added value of blue dye injection in selected patients. It is suggested that pre-operative lymphoscintigraphy imaging be considered as an integral part of sentinel lymph node mapping in surgical oncology. Detailed results of lymphoscintigraphy images should be provided for surgeons prior to surgery, and in case the sentinel lymph nodes are not visualized, use of blue dye for sentinel node mapping should be encouraged.

## Introduction

Sentinel node mapping, as a useful method for regional lymph node staging, minimizes the morbidity associated with lymph node dissection in patients with solid tumors.

The concept of sentinel node mapping relies on an orderly and predictable pattern of lymphatic flow from tumors. Sentinel nodes are the first lymph nodes in the lymphatic drainage system of tumors, and can be considered as surrogates for regional lymph nodes, regarding the pathological involvement (1, 2).

Two conventional methods for sentinel lymph node mapping are injection of radiotracer and blue dye. A combination of radiotracer and blue dye injection for lymphatic mapping is found to increase the detection rate and decrease the false-negative rate of sentinel node biopsy (3). However, several authors have proposed a more restricted use of blue dye injection due to potential life-threatening complications, associated with this method (4-6).

## Case report

A 56-year-old female patient with a histologically proven squamous cell carcinoma (2 cm in diameter) of uterine cervix was scheduled for sentinel node mapping in the nuclear medicine department of our institute. The patient was planned to undergo total hysterectomy and bilateral salpingo-oophorectomy.

Eighteen hours before the surgery, the patient received two intra-cervical injections of ^99m^Tc- Phytate at 3 and 9 o’clock positions (1 mCi/0.1 cc for each injection). Lymphoscintigraphy images with anterior-posterior and lateral views were obtained 30 min after the injection, using a dual-head variable-angle gamma camera (ECAM, Siemens), as previously specified (7). The gamma camera was equipped with low-energy high-resolution collimator and images were taken by ^99m^Tc photopeak and scatter photopeaks (for imaging the hue of the patient) (1 image/5 min).

**Figure 1:**
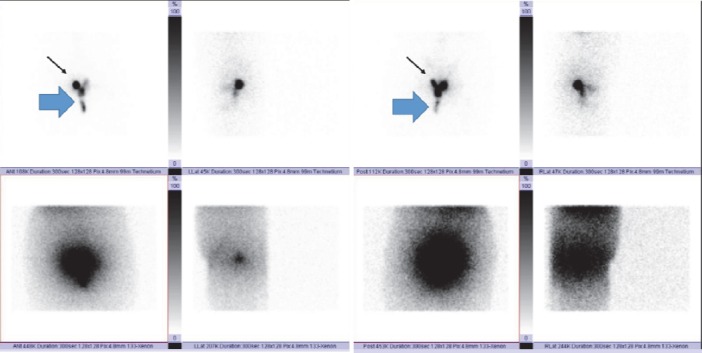
Early lymphoscintigraphy images of the patient. The original images are shown in the upper row and the scatterograms of the patient's hue in the lower row. Note minimal activity in the cervix (black arrows) and extension of radiotracer in the vagina (blue large arrows). No sentinel node could be visualized in the pelvis

Lymphoscintigraphy images showed improper injection of radiotracer in the cervix, as most of the radioactivity was visible in the vagina (Figure 1); also, no sentinel nodes were seen on the lymphoscintigraphy images.

**Figure 2:**
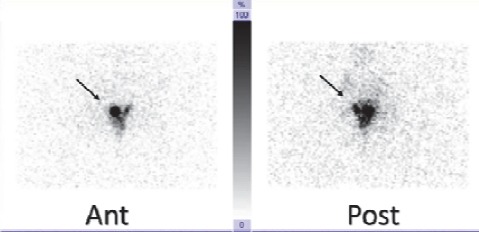
Delayed anterior/posterior lymphoscintigraphy images of the patient. No sentinel node is visible. Arrows are the injection sites in the cervix

The patient refused to undergo any further radiotracer injection. Pre-operative lymphoscintigraphy images also showed the same findings without any visible sentinel lymph nodes (Figure 2). The patient received two intra-cervical injections of methylene blue (0.5 cc/injection), and lymphatic mapping was performed using a gamma probe (EUROPROBE, France) and blue dye technique.

Two blue sentinel nodes were identified intra-operatively in the right and left obturator regions. However, no hot sentinel nodes were detected by the gamma probe. Frozen section examination of the sentinel nodes was negative for pathological involvement, and no pelvic lymph node dissection was performed.

## Discussion

The importance of blue dye technique in sentinel node mapping has been demonstrated by many researchers (5, 8). The rationale behind blue dye technique is to decrease false-negative rate of sentinel node mapping and increase the intra-operative detection of sentinel nodes.

However, addition of blue dye is associated with some risks including life-threatening anaphylactic reactions (6, 9, 10). Therefore, some authors have proposed a more restricted use of blue dye in sentinel node mapping (11). In an important study, Derossis et al. reported that the marginal benefit of blue dye injection in breast cancer patients decreases as the experience of surgeons increases (12). Another study by Sadeghi et al. reported similar findings, and showed the marginal benefits of blue dye technique in case of sentinel node visualization on lymphoscintigraphy images (4).

The present case shows the importance of lymphoscintigraphy imaging as an integral part of sentinel node mapping. Lymphoscintigraphy images indicated the inappropriate injection of radiotracer (in our case, failure to inject the air bubble behind the radiotracer in syringes), and the surgeon was informed about the results before the surgery. Blue dye technique was successful for lymphatic mapping and the patient was spared pelvic lymph node dissection.

Therefore, non-visualization of sentinel nodes on lymphoscintigraphy images should be reported to surgical oncologists, and use of blue dye technique should be promoted in similar clinical situations.
